# PLX038A, a long-acting SN-38, penetrates the blood-tumor-brain-barrier, accumulates and releases SN-38 in brain tumors to increase survival of tumor bearing mice

**DOI:** 10.1038/s41598-024-64186-2

**Published:** 2024-06-19

**Authors:** Jinkyu Jung, Eric L. Schneider, Wei Zhang, Hua Song, Meili Zhang, William Chou, Niranjan Meher, Henry F. VanBrocklin, Mary Helen Barcellos-Hoff, Tomoko Ozawa, Mark R. Gilbert, Daniel V. Santi

**Affiliations:** 1 Neuro-Oncology Branch, Center for Cancer Research, National Cancer Institute, National Institutes of Health, Bethesda, MD USA; 2 ProLynx, Inc, 135 Mississippi Street, San Francisco, CA USA; 3 Department of Radiation Oncology, University of California San Francisco, San Francisco, CA USA; 4 Department of Radiology and Biomedical Imaging, University of California San Francisco, San Francisco, CA USA; 5 Brain Tumor Center, Department of Neurological Surgery, University of California San Francisco, San Francisco, CA USA

**Keywords:** Drug development, CNS cancer

## Abstract

Central nervous system tumors have resisted effective chemotherapy because most therapeutics do not penetrate the blood-tumor-brain-barrier. Nanomedicines between  ~ 10 and 100 nm accumulate in many solid tumors by the enhanced permeability and retention effect, but it is controversial whether the effect can be exploited for treatment of brain tumors. PLX038A is a long-acting prodrug of the topoisomerase 1 inhibitor SN-38. It is composed of a 15 nm 4-arm 40 kDa PEG tethered to four SN-38 moieties by linkers that slowly cleave to release the SN-38. The prodrug was remarkably effective at suppressing growth of intracranial breast cancer and glioblastoma (GBM), significantly increasing the life span of mice harboring them. We addressed the important issue of whether the prodrug releases SN-38 systemically and then penetrates the brain to exert anti-tumor effects, or whether it directly penetrates the blood-tumor-brain-barrier and releases the SN-38 cargo within the tumor. We argue that the amount of SN-38 formed systemically is insufficient to inhibit the tumors, and show by PET imaging that a close surrogate of the 40 kDa PEG carrier in PLX038A accumulates and is retained in the GBM. We conclude that the prodrug penetrates the blood-tumor-brain-barrier, accumulates in the tumor microenvironment and releases its SN-38 cargo from within. Based on our results, we pose the provocative question as to whether the 40 kDa nanomolecule PEG carrier might serve as a “Trojan horse” to carry other drugs past the blood-tumor-brain-barrier and release them into brain tumors.

## Introduction

Tumors of the central nervous system (CNS) are among the deadliest forms of cancer and have defied effective chemotherapy. A primary hurdle for treating these cancers is delivering drugs through the modified blood–brain-barrier in the tumor vasculature—referred to as the blood-tumor-brain-barrier (BTBB)^1,2^. The use of nanomedicines (NM) could have a significant impact as an approach for drug delivery to brain tumors. Generally, NMs are molecules or particles between  ~ 10 and 100 nm in diameter, and can accumulate in tumors by the enhanced permeability and retention (EPR) effect^3,4^. The EPR effect results from the leaky vasculature of tumors that allow NMs to penetrate, and the poor lymphatic clearance that helps retain them in tumors. While the EPR effect is a well-established phenomenon, it remains controversial as to whether it can be exploited for treatment of brain tumors.

Macromolecular prodrugs may be effective against brain-tumors by either releasing their drug cargo systemically which then penetrates the BTBB to exert anti-tumor effects, or by directly penetrating the BTBB as prodrugs and releasing their cargo within the tumor. However, differentiating transport of a systemic drug to the brain tumor vs prodrug penetration of tumors may require diverse approaches and be challenging to accomplish.

In this context, there is evidence that certain nanocarrier prodrugs of the topoisomerase 1 (TOP1) inhibitor CPT-11 (irinotecan) may function by the prodrug tumor-penetrating mechanism. Here, the prodrug would penetrate the tumor, CPT-11 would be released, and then converted to its active metabolite SN-38 by carboxylesterase 2 (CE2). To date, CPT-11 prodrug-penetrating NMs that have been studied in brain tumors include NKTR-102—a 20 kDa ~ 12 nm 4-arm PEG-CPT-11 conjugate—that releases CPT-11 and is effective in treatment of intra-cranial MDA-MB-231 breast cancer^5^, and Onyvide—a ~ 100 nm liposome containing CPT-11—that is effective in both intra-cranial MDA-MB-231 breast cancer^6^ and U87MG glioblastoma^7^. These CPT-11 prodrugs appear to accumulate in brain tumors, have long intra-tumoral half-lives, and slowly release their CPT-11 cargo within the tumor to be converted to the potent TOP1 inhibitor, SN-38.

While the studies of CPT-11 prodrugs in brain tumors are promising, they share a common shortcoming. Carboxyesterase 2 (CE2) is required for metabolism of CPT-11 to SN-38, and that conversion primarily occurs in the liver^8^. It is implausible that CPT-11 is released from the prodrug in the tumor, then travels to the liver to form SN-38 which then returns to the tumor. So, it can be concluded that CPT-11 must be converted to the active SN-38 metabolite by CE2 in the tumor to be effective. However, tumor CE2 is not uniformly found in and not uniform in brain tumors^9–11^. Although intra-tumoral CE2 represents an uncontrollable variable that would affect efficacy, the variable can be eliminated by simply using prodrugs of SN-38 rather than CPT-11. To our knowledge, there are no direct prodrugs of SN-38 currently being pursued for treatment of CNS tumors.

PLX038 is a long-acting prodrug of SN-38 composed of a 15 nm 4-arm 40 kDa PEG attached to four SN-38 moieties by linkers that slowly cleave to release the drug^12,13^. The SN-38 released from PLX038 has a low C_max_ and very long t_1/2_ of about 5 days in humans—about tenfold longer than the SN-38 released from CPT-11. And, it has been shown in studies of Onivyde in mouse and man that efficacy is associated with the duration SN-38 remains above a threshold, while neutropenia is associated with SN-38 C_max_^9,14^. The 4-branched PEG_40kDa_ component of PLX038 provides a neutral, highly flexible, long half-life NM with a diameter of  ~ 15 nm in accord with ideal properties for NM tumor accumulation by EPR^4^. For preclinical studies, PLX038A was developed which is a slightly modified version of PLX038 that simulates its pharmacokinetics in humans to those in mice^15^. Earlier studies confirmed that PLX038A penetrates pores of tumor vasculature, accumulates and is retained in the tumor microenvironment for long periods^16^. Hence, we considered it possible that our PEGylated prodrugs of SN-38 might also accumulate in brain tumors—especially in the core of the tumor where there is abundant leaky vasculature^17^—and be candidates for treatment of brain tumors. In the present work, we show that PLX038A is highly effective for treatment of intracranial breast cancer and glioblastoma in mice. We also provide evidence that the macromolecular prodrug penetrates the BTBB and releases SN-38 within the tumor environment. Finally, we propose the intriguing possibility that the 40 kDa PEG carrier of PLX038 with a releasable linker might serve to transport other drugs past the BTBB and into brain tumors.

## Results

### Effect of PLX038A on SC and IC MDA-MB-436 TNBC

We examined the tumor growth inhibition properties of PLX038A on BRCA1-deficient MDA-MB-436 triple negative breast cancer (TNBC) xenografts. This tumor was chosen for study because its deficiency of BRCA1^18^ confers high sensitivity to TOP1 inhibitors^19,20^ including the PLX038A prodrug^15^, and because it represents an intracranial tumor model of TNBC metastatic breast cancer^21^.

**Figure 1:**
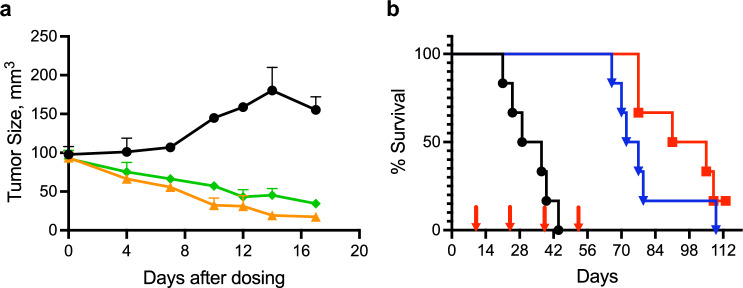
Effect of PLX038A on subcutaneous and intra-cranial MDA-MB-436. (**a**) MDA-MB-436 implanted subcutaneously (n = 3) and treated with a single dose of PLX038A at 30 μmol/kg () or 60 μmol/kg () or untreated (); median values ± median average deviation (MAD). (**b**) MDA-MB-436 implanted intracranially in 6 mice and treated with 30 μmol/kg () or 60 μmol/kg () PLX038A on days 10, 24, 38 and 52 (arrows) or untreated () (both doses, *p* = 0.0005). *p*-values were calculated by two sided log-rank test.

First, we studied the sensitivity of subcutaneous MDA-MB-436 tumors to PLX038A to ensure the tumor was intrinsically sensitive to the prodrug and to guide dosing of corresponding intracranial (IC) tumors. Figure 1a shows the growth curves of MDA-MB-436 xenografts in mice treated with single doses of 30- or 60 μmol/kg of PLX038A. As shown, both single doses caused significant tumor regression for ≥ 14 d (*p* =  < 0.0001). Hence, as an extra-cranial tumor, MDA-MB-436 is quite sensitive to PLX038A. Note that we refer to the PLX038A administered as μmol/kg where μmol refers to the amount of SN38 so that 1 μmol is 0.4 mg SN-38 or 10.4 mg PEG-SN-38 (PLX038A).

Next, we examined the effects of PLX038A on the survival of mice harboring intracranial MDA-MB-436 tumors. The in vivo sensitivity of SC tumors informed us that single doses of either 30- or 60 μmol/kg PLX038A could suppress tumors for ≥ 14 d (*p* = 0.0005). We reasoned that, because of the BTBB, inhibition of the IC tumor would require more intense dosing than in SC tumors so we treated IC tumor-bearing mice with a Q2Wk (every 2 weeks) schedule of PLX038A at these doses. As shown in Fig. 1b, mice with IC tumors were treated with four Q2Wk 30- or 60 μmol/kg doses of PLX038A starting on d 10. Then, PLX038A was withheld to see if the tumor would regrow in the absence of treatment. Mice treated with vehicle control had a median survival time (MST) of 29 days. Over the duration of treatment, all mice survived with no signs of disease. However, after the last dose at d 52, mice treated with the lower 30 μmol/kg dose of PLX038A started to succumb and showed a MST of 72 days that was 43 days longer than control. All but one mouse died by day 79. Mice treated with the more intense 60 μmol/kg dose of PLX038A showed a MST of 91 d and one mouse survived the entire 112 d observation period. Hence, at the Q2Wk 30- and 60 μmol/kg doses used, PLX038A resulted in  ~ 2.5- and  ~ 3.1-fold increases, respectively, in survival of mice bearing IC MDA-MB-436; notably, the survival of mice treated with either dose exceeded the twofold benefit reported for 50 mg NK102 (25 μmol SN38)/kg^6^ or 1.4-fold benefit from 50 mg/kg liposomal irinotecan^6^ in MDA-MB-231. Taken together, the results show that growth of IC MDA-MB-236 can be suppressed by Q2Wk dosing of PLX038A.

### Effect of PLX038A on SC and IC U251 glioblastoma

To study the efficacy of PLX038A on an orthotopic glioblastoma, we chose the U251 model because of its high in vitro sensitivity to SN-38 (EC_50_ ~ 5 nM).

**Figure 2:**
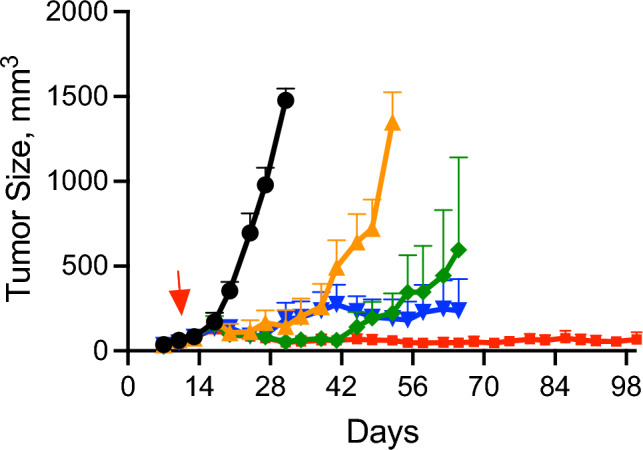
Effect of PLX038A on subcutaneous U251. Tumor volume vs time of U251 implanted subcutaneously in mice (n = 8) and treated with single doses of PLX038A at 15 μmol/kg () or 60 μmol/kg (), 8 QWk doses at 15 μmol/kg () or 60 μmol/kg (), or untreated (); data is median of tumor volumes ± MAD. *p* =  < 0.0001 for all doses vs vehicle. *P*-values were calculated by 2-way ANOVA.

To ensure U251 was sensitive to PLX038A in vivo, we first studied the response of SC tumors to the prodrug, eliminating barriers due to the BTBB. Single doses of 15 μmol/kg and 60 μmol/kg gave similar inhibition of U251 growth until ~ 11 d when median of tumors treated with the lower dose began reemerging; tumors treated with 60 μmol/kg were suppressed until ~ 25 d after dosing (Fig. 2). Since each single dose tested inhibited growth for ≥ 7 d, we treated the tumor-bearing mice with the same doses of PLX038A on a QWk schedule. Here, growth suppression by QWk 15 μmol/kg—dose-equivalent to the Q2Wk 30 μmol/kg used with IC MDA-MB-436—lasted significantly longer than single doses (*p* =  < 0.0001). The more intense QWk 60 μmol/kg dose gave complete inhibition for ≥ 80 d (*p* =  < 0.0001). Based on the duration of tumor suppression of single doses, it is likely that the intensity of repeat dosing could be reduced. Taken together, these results show that U251 is intrinsically sensitive to PLX038A in vivo, and that a QWk repeat-dosing schedule can maintain SC tumor growth suppression for long periods.

**Figure 3:**
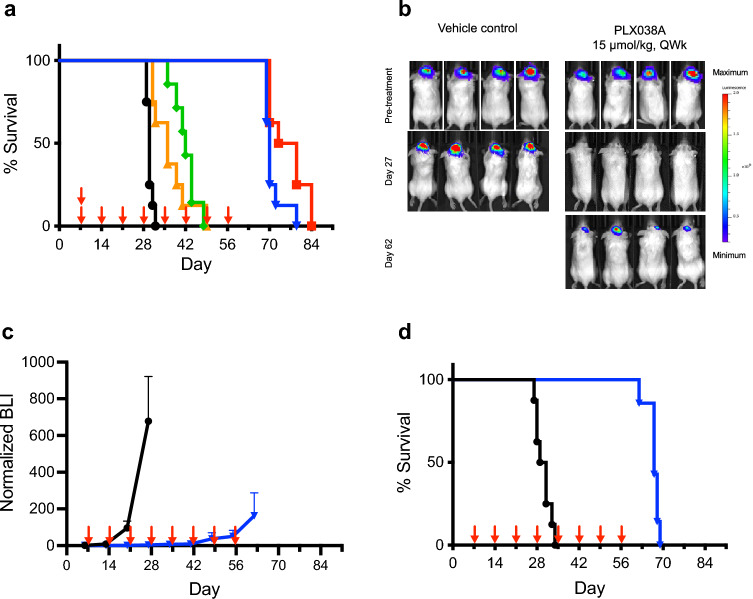
Effect of PLX038A on intracranial U251. (**a**) Survival curve of mice (n = 7) with U251 implanted intracranially and treated on day 7 with a single dose of PLX038A (single arrow) at 15 μmol/kg (, *p* = 0.001) or 60 μmol/kg (, *p* = 0.0001); 8 QWk doses at 15 μmol/kg (, *p* =  < 0.0001) or 60 μmol/kg (, *p* =  < 0.0001) on days 7, 14, 21, 28, 35, 42, 49 and 56 (arrows); or untreated (). (**b**) Representative bioluminescence images in U251 Luc before treatment, at d 27 and at d 62 after initiation of treatment with 15 μmol/kg PLX038A. (**c**) Growth curve by luminescence of U251 Luc tumors implanted intracranially and treated on day 7 with 8 QWk doses at 15 μmol/kg () (arrows) or untreated () (PLX038A n = 7, untreated n = 8). Data is median BLI ± median average deviation. (**d**) Survival curve of mice (PLX038A n = 7, untreated n = 7) with U251 Luc implanted intracranially and treated on day 7 with 8 QWk doses (indicated by arrows) at 15 μmol/kg (, *p* = 0.0004) or untreated (). The MST for U251 Luc was identical to WT U251 at 30 d for the untreated and 68 d for treated animals. Endpoints for survival curves were death or euthanization when mice acquired severe neurological symptoms. *p*-values were calculated by two sided log-rank test.

We next examined the effects of PLX038A on survival of mice harboring intracranial U251 tumors (Fig. 3). Here, the SC in vivo sensitivity experiments informed us that a single dose of PLX038A would likely not cause long-duration growth suppression of IC tumors, but that weekly dosing should be effective. Figure 3a shows the effect of 15- and 60 µg/kg PLX038A on survival of mice with intracranial U251 tumors both as a single dose or eight QWk doses. Single doses of 15- and 60 µmol/kg PLX038A increased the MST from 30 d in the control to 36 d (*p* = 0.001) and 42 d (*p* = 0.0001), respectively, corresponding to modest 1.2- and 1.4-fold increases. In contrast, QWk 15- and 60 µmol/kg PLX038A showed significantly increased MSTs of 70 d (*p* =  < 0.0001) and 73 d (*p* =  < 0.0001), respectively, a 2.3- and 2.4-fold increase over control.

We also generated intracranial tumors using luciferase modified human glioblastoma U251 cells, and treated tumor-bearing mice with QWk 15 µmol/kg PLX038A. As shown in Fig. 3b, mice possessed large IC tumors at the outset of treatment that were no longer visible 27 d after treatment initiation, but returned after drug withdrawal (all mice in study presented in Figure S1C). With 8 QWk doses at 15 μmol/kg, measurements of luciferase vs time showed complete growth inhibition up to the last dose at d 56 (Fig. 3c) and the survival curves in Fig. 3d closely resembled that of U251 without luciferase (Fig. 3a). After cessation of treatment, tumors resumed exponential growth and mice died with MST at 70 d. Replicate experiments were in excellent agreement (Fig. S1). Taken together these results show that sustained exposure to PLX038A suppressed intracranial U251 tumor growth and increased survival rate; however, tumor growth resumed after drug withdrawal.

**Figure 4:**
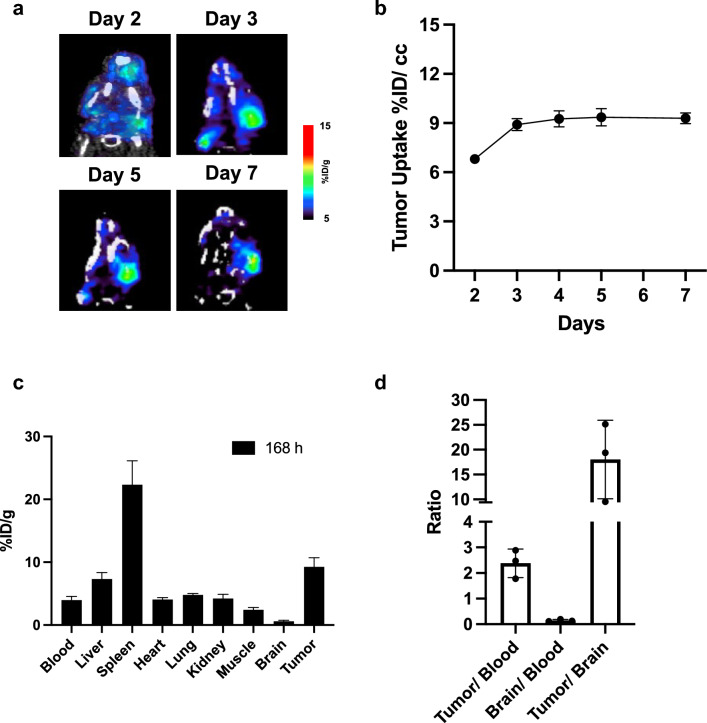
PET imaging of mice bearing IC U251 glioblastoma with ^89^Zr-PEG_40kDa_(DFB)_4_. (**a**) Temporal mPET/CT maximum intensity projection images of the same mouse bearing IC U251 glioblastoma. (**b**) Time-activity curve of tumor ROIs showing mean ± SE. Not all tumors showed uptake above background at d 1, and data starts at day 2. (**c**) Ex vivo biodistribution of ^89^Zr-PEG_40kDa_(DFB)_4_ in tumor and various tissues at d 7. (**d**) Selected tumor and tissue ratios at d 7, showing mean ± SE.

Finally, we studied the uptake of the PLX038A surrogate, [^89^Zr]PEG_40kDa_(DFB)_4_, using μPET/CT imaging in mice bearing U251 glioblastoma. Mice with U251 glioblastoma verified by bioluminescence (Fig. S1) were treated and sample images are reproduced in Fig. 4a (and Fig. S2). As shown in the time-activity curve in Fig. 4b, the agent concentrates to  ~ 10%ID in IC tumors by 3 d—similar to that in SC tumors^16^—and then remains constant for ≥ 7 d. Since PEG_40kDa_ has a systemic elimination t_1/2_ of only  ~ 1- to 2 d, we conclude that once in the tumor, [^89^Zr]PEG_40kDa_(DFB)_4_ has a very long retention time. On d 7, 3 mice were euthanized, major organs were collected and biodistribution of [^89^Zr]PEG_40kDa_(DFB)_4_ was determined ex vivo by gamma counting (Fig. 4c). We found some tumors adjacent and adhering to the brain inside the skull, likely a result of tumor cell reflux upon inoculation. As shown, the %ID uptake at 7 d was highest in spleen and tumor, with tumor containing 9.3 ± 2.5%ID/g, similar to accumulation of [^89^Zr]PEG_40kDa_(DFB)_4_ in SC tumors^16^. Very little signal was present in brain tissue yielding a high tumor/brain ratio, 18.0 ± 7.9; the tumor/blood was a high 2.5 (Fig. 4d). Hence, the PEG_40kDa_(DFB^89^Zr)_4_ does not accumulate in normal brain tissue, but accumulates to high levels in IC tumors.

## Discussion

The objectives of the present work were to determine whether PLX038A is effective in treating preclinical models of primary and metastatic brain tumors—U251 glioblastoma and MDA-MB-436 TNBC—and, if so, to determine whether the prodrug serves as a systemic source of SN-38 or penetrates the BTBB and releases SN-38 in the tumor microenvironment.

Previous reports suggested that PEGylated and liposomal prodrugs of CPT-11 accumulate in brain tumors, and then release their drug cargo to inhibit growth of tumors^5–7^. However, with these prodrugs the CPT-11 released must be converted to active SN-38 by tumor CE2, which presents an uncontrollable variable that alters the drug availability and efficacy. Here, we completely avoided this problem by using PLX038A as a direct prodrug of SN-38 which was much more efficacious than CPT-11 or the CPT-11 prodrugs.

We first studied the sensitivity of SC implanted MDA-MB-436 and U251 tumors to PLX038A to ensure the tumors were susceptible to the prodrug in the absence of a BTBB. One treatment with 30- and 60 μmol/kg PLX038A informed us that the SC tumors were sensitive, and on the duration of tumor suppression by a single dose. With MDA-MB-436 biweekly treatment of either dose was quite effective, and with U251 weekly treatment with as low as 15 μmol/kg suppressed tumor growth for long periods. These results confirmed that both tumors are intrinsically sensitive to PLX038A in vivo, and assisted in designing effective dosing regimens for corresponding IC tumors.

Next, we showed that IC-implanted MDA-MB-436 and U251 were also highly sensitive to PLX038A. Indeed, the same dosing-equivalent schedule to a low 15 μmol/kg/wk—administered as either Q2Wk 30 μmol/kg for MDA-MB-436, or QWk 15 μmol/kg for U251—that was effective for SC tumors was likewise effective at suppressing IC tumor growth and increasing survival of hosts. Clearly, both IC MDA-MB-436 and IC U251 tumors are highly sensitive to the SN-38 from PLX038A.

The growth inhibition of IC TNBC and glioblastoma tumors by PLX038A could be impactful. The observation that PLX038A is effective in inhibiting extra- or intra-cranial BRCA1 deficient MDA-MB-436 may in itself be relevant. Since there are no chemotherapies for TNBC patients with brain metastases, PLX038 may be useful in treating patients with BRCA-deficient breast tumors that have metastasized to the brain. Also, the prodrug could act as a long-acting, localized DNA-damaging agent in IC tumors that could augment the effect of a BTBB-permeable PARP inhibitor on PARP inhibitor sensitive tumors. Importantly, since there are no effective drugs available to treat glioblastoma, translation of the PLX038 inhibition of IC glioblastoma to humans would have a major effect on future treatment of the disease.

Even though we achieved growth suppression of IC tumors for long periods, when dosing was discontinued tumor growth resumed with ensuing death of most mice. Hence, PLX038A suppresses tumor growth but does not cure animals of the tumor. It is now well established that, in addition to cytotoxic effects, TOP1 inhibitors have potent immunomodulatory effects including stimulation of the STING pathway to turn cold tumors to hot, and sensitization of tumors to checkpoint blockade, notably αPD1^22–26^; additionally, TOP1i cause immunogenic cell death^27,28^. It would be of interest to test PLX038A and αPD-1 in immunocompetent animal models to determine the efficacy of the chemoimmunotherapy combination in providing lasting cures. Because PLX038 is already in clinical trials, and many αPD-1 s are approved, such studies could be expeditiously translated to humans.

A most important issue is whether PLX038A releases SN-38 systemically which then penetrates the brain to exert anti-tumor effects, or whether the prodrug directly penetrates the BTBB and releases its SN-38 cargo within the tumor. Our data strongly supports a tumor-penetrating mechanism for PLX038A.

First, PLX038A is highly effective at increasing survival of mice bearing IC tumors, yet the maximal amount of SN-38 that can be provided by the therapeutically effective 15 μmol/kg/wk PLX038A is insufficient to account for this efficacy. Consider, for example, that weekly administration of 85 μmol/kg CPT-11—forming  ~ 35 μmol of SN-38^29^—to mice bearing IC MDA-MB-231 tumors did not prolong survival^5^. Likewise, weekly dosing of 106 μmol/kg CPT-11—forming a very high  ~ 45 μmol of SN-38—to mice bearing IC U87MG glioblastoma gave only a modest increase in survival^7^; yet, only 15 μmol/kg/wk of PLX038A causes durable growth inhibition and a high increase in survival of both MDA-MB-236 and U251 brain tumors. Hence, the SN-38 derived from systemic release of SN-38 from PLX038A appears to be insufficient to cause the observed antitumor effects.

Second, ^89^Zr-PEG_40kDa_(DFB)_4_—a PET imaging surrogate for PEG_40kDa_(SN-38)_4_—concentrates in the IC U251 tumor to a high  ~ 10%ID uptake within  ~ 3 days and is retained for over 7 days without any uptake in normal brain tissue. This is similar to the  ~ 10%ID uptake and retention of ^89^Zr-PEG_40kDa_(DFB)_4_ in SC xenografts, again without measurable uptake in brain^16^. Indeed, the 4-branched PEG_40kDa_ carrier provides a neutral, flexible, long half-life NM with a diameter of  ~ 15 nm that has ideal properties for tumor accumulation by EPR^4^. Shortly, a trial will determine if the ^89^Zr-PEG_40kDa_ surrogate will concentrate in human tumors and serve to predict tumor response to prodrugs such as PLX038. Taken together, the evidence indicates that PLX038A does not pass the BBB but directly penetrates the BTBB, accumulates in the tumor and releases its SN-38 cargo from within.

At first, it may seem perplexing that PLX038A can be so effective at inhibiting IT tumor growth in spite of the facts that the extra-cellular pH of tumors such as U251 can be as low as pH 6.5^30^, and that release of PLX038A is inhibited by acidic media. However, the high accumulation and long retention of the NM in the tumor may counteract the effect of slow drug release by increasing the duration of tumor exposure to SN-38. Indeed, the anti-tumor efficacy of the SN-38 prodrug Onivyde is associated with a longer duration of SN-38 above a critical threshold^9,14^. Hence, the reduction of cleavage rate of PLX038A by an acidic tumor microenvironment may serendipitously prolong exposure to SN-38 and enhance rather than diminish its antitumor activity.

In summary, the SN-38 prodrug PLX038A caused high tumor growth inhibition of model intracranial TNBC and glioblastoma tumors and significantly increased survival of tumor-bearing mice. However, the BTBBs in human tumors are quite different than those in preclinical models, and the potential utility of the prodrug needs to await results of clinical trials. Since the parental PLX038 is already in clinical trials, it would be a straightforward path to translate these experiments to trials on human brain tumors; indeed, a trial of PLX038 in MYC-amplified CNS tumors has been initiated (NCT06161519). Additionally, a trial is planned for ^89^Zr-PEG_40kDa_(DFB)_4_—the PET imaging surrogate for PEG_40kDa_(SN-38)_4_—to determine whether the nano-particle can penetrate the BTBB of human tumors. Further, since TOP1 inhibitors activate the STING pathway and may turn cold tumors hot and visible to immune cells, going forward it would be of high interest to investigate combinations of PLX038 with checkpoint inhibitors. Finally, the preponderance of evidence indicates that the prodrug penetrates the BTBB, accumulates in the tumor microenvironment, and slowly releases the SN-38 cargo from within. This raises the provocative possibility that the 4-arm 40_ kDa_ PEG scaffold used for PLX038 with a releasable linker could serve as a “Trojan horse” to carry other drugs past the BTBB and into brain tumors.

## Methods

PLX038A was prepared as described and quantified by UV absorbance of the contained SN-38 using ε_310nm_ = 22,500 M^−1^ cm^−1^ at pH 7. Doses of PLX038A refer to the amount of SN-38 contained. Animal experiments performed at the University of California San Francisco were performed under a protocol reviewed and approved by the Institutional Animal Care and Use Committee (IACUC) at the University of California San Francisco. Animal experiments performed at the National Institutes of Health (NIH) were performed under procedures outlined in the NIH Guide for the Care and Use of Animals and approved by the Animal Care and Use Committee (ACUC) of the NIH. All studies were performed in accordance with ARRIVE guidelines. Unless otherwise noted, animals were euthanized by carbon dioxide inhalation followed by cervical dislocation. Detailed descriptions of materials and procedures are presented in the Supplementary Information (SI).

### Subcutaneous xenografts

Eight-week-old female immunodeficient mice were implanted in right flanks with MDA-MB-436 or U251 cells. Once the size of tumors reached ~ 100- to 200 mm^3^, mice were treated with specified doses of IP PLX038A or vehicle control. Tumor growth was measured 2- to 3- times weekly using vernier calipers and tumor volumes were calculated and plotted vs time.

### Intracranial xenografts

Eight-week-old female mice were injected intracranially with MDA-MB-436 or U251 cells, or U251 cells tagged with luciferase. Mice were treated with specified doses of IP PLX038A or vehicle control. Survival endpoints were recorded as animal death or when they were sacrificed if they had severe neurological signs or a body weight loss of more than 15%. For bioluminescence imaging, luciferin was administered via IP injection and examined for tumor bioluminescence with an IVIS Lumina imaging station; regions of interest were recorded as photons per second per steradian per cm^2^.

### PET imaging of glioblastoma IC xenograft

^89^Zr radiolabeling of the tetra-PEG nanocarrier and purification of the [^89^Zr]PEG_40kDa_(DFB)_4_ conjugate was performed exactly as previously described^16^. The isolated activities were 139–146 MBq and radiolabeling yields ranged from 46- to 49 MBq/mg. Intracranial cell implants of U251 cells were visiualized by bioluminescence on d 10 and 15 post implantation. On day 17 [^89^Zr]PEG_40kDa_(DFB)_4_ was administered via tail vein (5.5- to 7.4 MBq in 100 μL/mouse), and serial *µ*PET/CT imaging data from 24- to 168 h post injection were acquired. Post 168 h *µ*PET/CT imaging, mice were euthanized by anesthetizing in 2% isoflurane followed by cervical dislocation. Blood and major organs were collected, weighed and analyzed.

### Supplementary Information

Supplementary Information.

## Data Availability

All study data are included in the article and/or in the SI.
