# Supplementary material for: PLX038A, a long-acting SN-38, penetrates the blood-tumor-brain-barrier, accumulates and releases SN-38 in brain tumors to increase survival of tumor bearing mice
Source: Sci Rep. 2024 Jun 19;14:14175. doi: 10.1038/s41598-024-64186-2 (PMC11187204; doi:10.1038/s41598-024-64186-2)
Supplement: Supplementary file 1 — Supplementary Information. [file 41598_2024_64186_MOESM1_ESM.docx]

**Supplementary information**

**PLX038A, a long-acting SN-38, penetrates the blood-tumor-brain-barrier, accumulates and releases SN-38 in brain tumors to increase survival of tumor bearing mice.**

Contents:

1. MDA-MB-436 BRCA-deficient TNBC inhibition by PLX038A

2. U251 glioblastoma tumor inhibition by PLX038A

3. PET imaging of PLX038A in U251 glioblastoma brain tumor

**––––––––––––––––––––––––––––––––––––––––––––––––––––––**

**1. MDA-MB-436 BRCA-deficient TNBC inhibition by PLX038A**

Animal experiments were performed under a protocol reviewed and approved by the Institutional Animal Care and Use Committee (IACUC) at the University of California San Francisco (UCSF). MDA-MB-436 cells were authenticated and tested negative for mycoplasma by Idexx Bioanalytics, Columbia, Missouri.

Subcutaneous xenografts. Eight-week-old female *Foxn1^nu^/Foxn1^nu^* athymic nude mice (Jackson Laboratory, Bar Harbor ME) were injected in the right flank with 3 x 10^6^  MDA-MB-436 cells suspended in 100 ml of 1:1 Cultrex BME (Trevigen/Bio-Techne Minneapolis, MN) and PBS. Once the size of tumors reached ~60-100 mm^3^, mice were randomly enrolled into groups and treated with a single intra-peritoneal dose of PLX038A at 30 μmol/kg or 60 μmol/kg, or vehicle control (isotonic acetate buffer, pH 5.0). Tumor growth was measured three times weekly using vernier calipers and tumor volume was calculated as (longer diameter×(shorter diameter ^2^))× 0.52. Animals were sacrificed if the tumor grew to 10x10mm in size or if they developed any systemic issues such as body weight loss of more than 15%.

Intracranial xenografts. Eight-week-old female *Foxn1^nu^/Foxn1^nu^* athymic nude mice (Jackson Laboratory, Bar Harbor ME) were injected stereotactically with 0.5×10^6^ MDA-MB-436 cells suspended in 5 ml of Hanks Balanced Salt Solution (HBSS) and 0.5% Fetal Bovine Serum (FBS) 10 days after implantation, the mice were randomly enrolled into groups and treated intra-peritoneally with PEGylated PLX038A at 30 μmol/kg or 60 μmol/kg or vehicle control (isotonic acetate buffer, pH 5.0). Mice in the were given three additional doses every two weeks after the initial dose at day 10 (i.e. 4 total doses). Animals were observed and weighed three times weekly. Endpoints were recorded as animal death or when they were sacrificed if they developed neurological signs such as head tilts, hunching/circling, or had a body weight loss of more than 15%. At established endpoints, mice are euthanized by carbon dioxide inhalation followed by cervical dislocation.

**2. U251 glioblastoma tumor inhibition by PLX038A**

Animal experiments were performed under procedures outlined in the National Institutes of Health (NIH) Guide for the Care and Use of Animals and approved by the Animal Care and Use Committee (ACUC) of the NIH.

A. Subcutaneous xenografts (data in text). Eight-week-old female nude mice were injected using a 27 gauge needle in the right flank with 5 x 10^6^ U251 cells suspended in 100 μL PBS. Once the size of tumors reached ~160 mm^3^ (day 17 post inoculation), mice were randomly allocated into four groups and treated with a single dose of PLX038A at 15 μmol/kg or 60 μmol/kg, 8 QWk doses at 15 μmol/kg or 60 μmol/kg on Day 17, 24, 31, 38, 45, 52, 59 and 66. Tumor growth was measured at least twice weekly using vernier calipers and tumor volume was calculated as (longer diameter×(shorter diameter ^2^))×(𝜋)/6). Events for survival analysis were endpoints used to euthanize mice following NCI ACUC approved guidelines. Once tumor size became 2 cm in any dimension, ulcerated, or was clearly inhibiting functioning (i.e. ambulation, eating, etc.) animals were euthanized by carbon dioxide inhalation followed by cervical dislocation.

Intracranial xenografts (data in text). Four- to 6-week-old SCID mice were injected stereotactically with 3×10^5^ cells for U251 or U251 tagged luciferase. After implantation, the mice were randomly allocated into each group represented in the text and treated intra-peritoneally with vehicle (isotonic acetate buffer, pH 5.0) or PLX038A. The size of orthotopic tumors was monitored using the bioluminescence channel of IVIS Spectrum. Endpoints for survival curves of mice bearing U251 were death or euthanization by carbon dioxide inhalation followed by cervical dislocation when they acquired severe neurological symptoms (gait abnormalities, hunching, lethargy, seizures, paralysis, circling, etc.).

Table 1. P value for intracranial xenograft survival vs time

B. Intracranial xenografts of U251 (replicate experiment of data in text).

Cell Culture. U-251 human glioblastoma cell lines were obtained from the Department of Neurological Surgery Tissue Bank at the University of California, San Francisco (UCSF) and experiments performed at the UCSF Brain Tumor Center Pre-clinical Therapeutic Testing Core. Cells were maintained as exponentially growing monolayers in complete medium (CMEM) consisting of Eagle’s minimal essential medium supplemented with 10% fetal calf serum and 1% non-essential amino acids. Cells were been modified by lentiviral infection for stable expression of firefly luciferase to enable in vivo bioluminescence imaging. Cells were cultured at 37^°^C in a humidified atmosphere containing 95% air and 5% CO_2_. Cells were harvested by trypsinization, washed once with Hanks’ Balanced Salt Solution (HBSS) and resuspended in HBSS for intracerebral implantation.

Intracranial xenografts**.** Five to six-week-old female athymic mice (nu/nu, homozygous; Envigo Laboratories, Livermore, CA), housed under aseptic conditions, received intracranial tumor cell injection, as approved by the University of California San Francisco Institutional Animal Care and Use Committee. In brief, mice were anesthetized by combination of intraperitoneal injection of a mixture containing ketamine (100 mg/kg) and xylazine (10 mg/kg), and inhalation of isoflurane, and then were injected with 3 mL of tumor cell suspension (300,000 cells total) into the right caudate putamen by free hand method.

The scalp was surgically prepped, and a skin incision ~10 mm in length was made over the middle frontal to parietal bone.  The surface of the skull was exposed so that a small hole was made 3.0mm to the right of the bregma and just in front of the coronal sure with a 25-gauge needle.  A 26-gauge needle attached to a Hamilton syringe was inserted into the hole in the skull.  The needle was covered with a sleeve that limits the injection depth to 3-4mm. Cell suspensions in 3 uL was injected very slowly (~1 uL/ minute) by free hand and then the needle was removed.  The skull surface was swabbed with hydrogen peroxide before the hole is sealed with bone wax to prevent reflux.  The scalp was closed with surgical staples.

Bioluminescence Monitoring of Intracranial Tumor Growth. For bioluminescence imaging (BLI), mice were anesthetized with inhalation of isoflurane, then administered 150 mg/kg of luciferin (D-luciferin potassium salt, Gold Biotechnology, St. Louis, MO) via intraperitoneal injection. Ten minutes after luciferin injection, mice were examined for tumor bioluminescence with an IVIS Lumina imaging station and Living Image software (Caliper Life Sciences, Alameda, CA), and regions of interest were recorded as photons per second per steradian per square cm (Ozawa James. J Vis Exp 13: pii:1989.doi: 10.3791/1986, 2010).

**C**

**Figure S1.** Effect of PLX038A on intracranial U251 Luc. A) Survival curve of mice (n=8) with U251 Luc implanted intracranially and treated on day 7 with 8 QWk doses of PLX038A at 15 μmol/kg (▼) on days 8, 15, 22, 28, 36, 43, 50 and 57 after tumor implantation (indicated by arrows) or untreated (⚫). MST for U251 Luc was 26 days and 67 days for the untreated and treated animals, respectively. Endpoints for survival curves of mice bearing U251 were death or euthanization when they acquired severe neurological symptoms. B) Luminescence of U251 Luc tumors implanted intracranially in panel A. C) BLI images of 7- to 8 mice with luciferase transfected IC U251 at various times.

**3. PET imaging of PLX038A in U251 glioblastoma IC xenograft**

^89^Zr Radiolabeling of PEG: ^89^Zr radiolabeling of the tetra-PEG PEG nanocarrier was carried out following a previously reported protocol (Beckford Vera, Mol Cancer Ther, 19, 673, 2020). Briefly, ^89^Zr-oxalate (7 μL, ~150 MBq) was neutralized with 7 μL of Na_2_CO_3_ (1 M), and 500 µL of NH_4_OAc (1 M) was added to the mixture. To this mixture, ~3 mg of PEG_40kDa_(DFB)_4_ conjugates in 60 µL DI water were added and incubated for 30 min at 25 ˚C. The radiolabeled product was purified using PD-10 size-exclusion desalting column (Fisher Scientific, Hampton, NH ) and eluting with saline. Silica gel impregnated glass microfiber chromatography paper (Neta Scientific, Hainesport, NJ) was used for instant thin layer chromatography (iTLC) and was developed with 50 mM EDTA solution to confirm radiolabeling purity. The isolated bound activities were 139-146 MBq. The radiolabeling yields ranged from 46.3-48.6 MBq/mg.

*In Vivo* PET Imaging and Biodistribution Studies: Intracranial cell implantation of U251 cells was visiualized on day 10 and 15 post implantation. On day 17 [^89^Zr]PEG_40kDa_(DFB)_4_ was administered via tail vein (5.5 - 7.4 MBq in 100 μL of saline per mouse). The study population of 4 mice were subjected to serial *µ*PET/CT imaging (Inveon, Siemens Medical Solutions, Malvern, PA) at 24 h, 48 h, 72 h, 96 h, 120 h, and 168 h post injection of [^89^Zr]PEG_40kDa_(DFB)_4_. The PET data were acquired for 20 min in list mode. The manufacturer's 2-D ordered subsets expectation maximization (OSEM) algorithm was used to reconstruct the data. The imaging data were then normalized to the injected activity to parameterize images to %IA/cc. The imaging data was processed in an open-source AMIDE software (http://amide.sourceforge.net/). Post 168 h *µ*PET/CT imaging, the mice were were euthanized (anesthetizing the mice in 2% isoflurane, followed by cervical dislocation) to collect blood through cardiac puncture, and major organs (liver, kidney, spleen, heart, lung, brain, muscle, and tumor) were collected. Blood and major organs were weighed and analyzed in an automated gamma counter (Hidex, Turku, Finland). The percent injected dose per gram of tissue (% ID/g) was determined by comparing to radioactive standards.


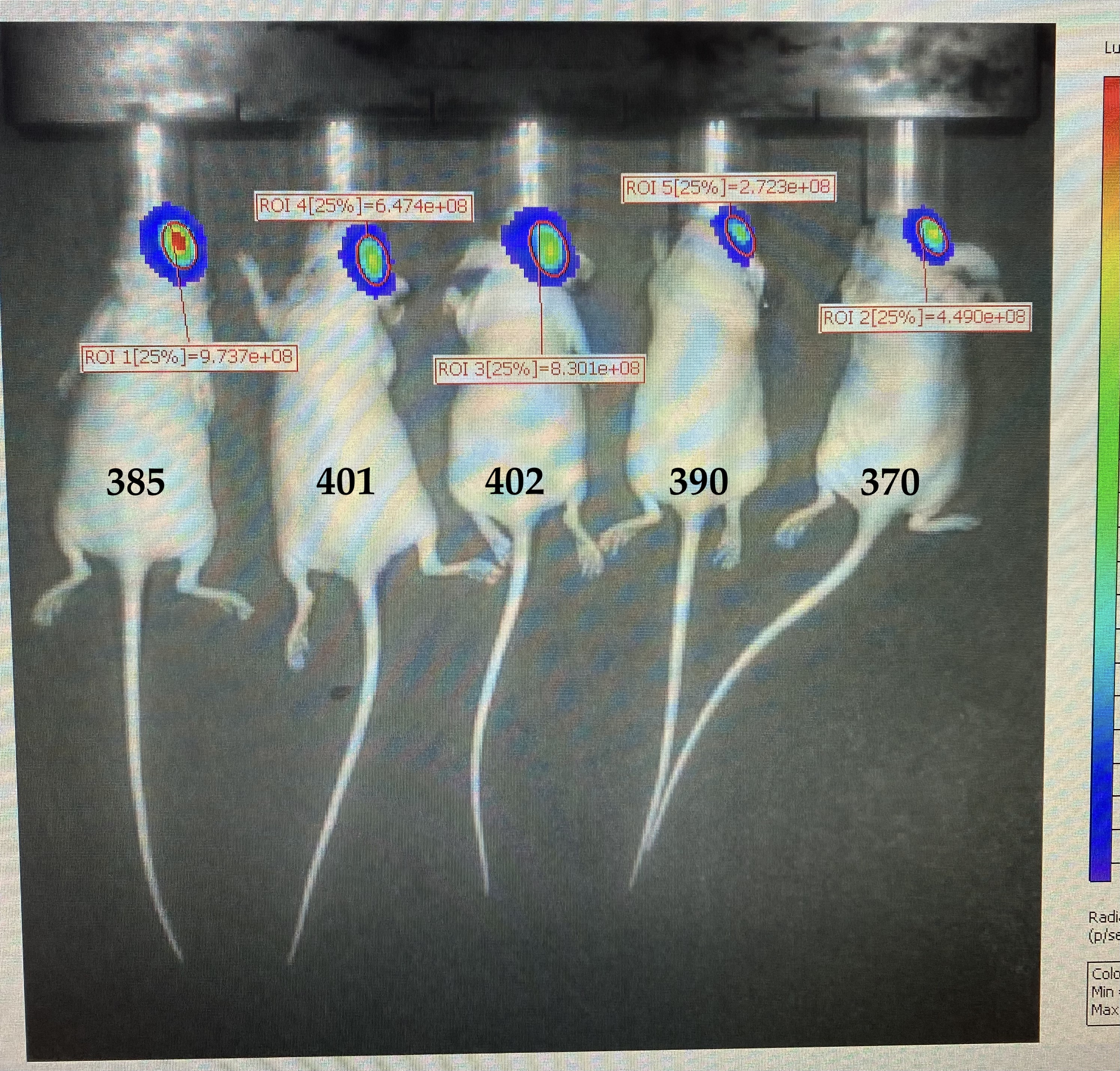


**Figure S2B.** BLI of mice with luciferase transfected IC U251 cells prior to PET imaging.

**Figure S3**. PET imaging of 4 mice bearing IC U251 tumors with ^89^Zr-PEG_40kDa_(DFB)_4_. Brain tumor images at 24h, 72h, 120h and 168h

**Figure S4**. Heart ROI vs time.
